# Different Strokes for Different Folks: The BodyMind Approach as a Learning Tool for Patients With Medically Unexplained Symptoms to Self-Manage

**DOI:** 10.3389/fpsyg.2018.02222

**Published:** 2018-11-13

**Authors:** Helen Payne, Susan Brooks

**Affiliations:** School of Education, University of Hertfordshire, Hatfield, United Kingdom

**Keywords:** medically unexplained symptoms, primary care, embodied approaches, adult learning, self-management, metaphor, symbol, group

## Abstract

Medically unexplained symptoms (MUS) are common in both primary and secondary health care. It is gradually being acknowledged that there needs to be a variety of interventions for patients with MUS to meet the needs of different groups of patients with such chronic long-term symptoms. The proposed intervention described herewith is called The BodyMind Approach (TBMA) and promotes learning for self-management through establishing a dynamic and continuous process of emotional self-regulation. The problem is the mismatch between the patient’s mind-set and profile and current interventions. This theoretical article, based on practice-based evidence, takes forward the idea that different approaches (other than cognitive behavioral therapy) are required for people with MUS. The mind-set and characteristics of patients with MUS are reflected upon to shape the rationale and design of this novel approach. Improving services for this population in primary care is crucial to prevent the iterative spiraling downward of frequent general practitioner (GP) visits, hospital appointments, and accident and emergency attendance (A&E), all of which are common for these patients. The approach derives from embodied psychotherapy (authentic movement in dance movement psychotherapy) and adult models of learning for self-management. It has been developed from research and practice-based evidence. In this article the problem of MUS in primary care is introduced and the importance of the reluctance of patients to accept a psychological/mental health referral in the first instance is drawn out. A description of the theoretical underpinnings and philosophy of the proposed alternative to current interventions is then presented related to the design, delivery, facilitation, and educational content of the program. The unique intervention is also described to give the reader a flavor.

## Introduction

Medically unexplained symptoms (MUS) are a thorny issue in primary care. Despite the differing nomenclature, the recent DSM-5 terms it as somatic symptom disorder (SSD) but is yet to achieve general usage. Many general practitioners (GPs) appear to reliably recognize MUS without the need for standardized assessments (Rasmussen et al., 2008). This population present with many, various and nebulous physical and psychological ailments (Rosendal et al., 2005) and constitute more than 25% of all new hospital and GP appointments (Fink et al., 1999; Reid et al., 2001). In England MUS has been estimated to cost £3 billion in 2008–2009 rising to £18 billion if loss of productivity, benefits and quality of life are accounted for Bermingham et al. (2010).

The increasing aging population, longer life span and higher number of people living with long term conditions is becoming a burden on an already over-stretched health service dealing with acute care as a priority. There is a move away from the passive patient to a more active role and involvement in line with the reality of a chronic, condition where day-to-day responsibility for disease management shifts from health care professionals to the individual patient. The United Kingdom (UK) Expert Patient initiative and the National Health Service (NHS) Direct adopts this view. So self-management becomes imperative.

Supporting patients with chronic conditions such as MUS to self-manage involves patients learning self-assurance, knowledge, and skills (Kroenke and Swindle, 2000; Wagner et al., 2001), “Medical care then must assure that persons with chronic illness have the confidence and skills to manage their condition” (ibid, 2).

Mental health interventions such as cognitive behaviour therapy (CBT) expect the patient with MUS to adapt to the intervention as opposed to the intervention being designed around the patient profile and their mind-set. It is vital to understand the profile of such complex patients to engage them in an intervention, some current interventions do not address this issue sufficiently. Consequently, patients with MUS may only engage in CBT for a short time, or not at all. The fact they do not attend is not reported, however, and this is true for both research studies and service delivery: “Most patients were studied after accepting referral for mental health treatment and these are only a fraction of all MUS patients” (Kroenke and Swindle, 2000, p. 211) The intervention described herewith caters for the larger number of MUS patients who do not attend or engage with CBT and has been designed with the patient profile in mind, in particular their mind-set. Patients have a belief in a physical cause and see a referral to CBT as a mental health concern with its associated stigma and a rejection of the legitimacy of their physical symptoms (Edwards et al., 2010).

Edwards et al. (2010, p. 209) state MUS are a “clinical and social predicament, which includes a broad spectrum of presentations, difficulty accounting for symptoms based on known pathology.” They go on to say this definition avoids the challenge of having to choose either an organic or a psychological explanation enabling a biopsychosocial treatment to address both hypotheses at the same time.

There are difficulties for the GP in diagnosing the symptom/s based on known pathology resulting in many appointments to specialists in hospitals. Furthermore, GPs are sometimes frustrated by the lack of a diagnostic category to provide them with the guidance required to deliver the most appropriate treatment. They can feel their hearts sinking when they see the patient again for the same symptom/s after so many visits and referrals. As a result, patients become known as “heart sink” patients (O’Dowd, 1988). This is an interesting label as it is the GP’s heart that sinks, but the patient that acquires the label. It may be that the GP inadvertently communicates their feelings of inadequacy and frustration to the patient which in turn exacerbates the patient’s symptoms and lack of agency. Clearly GPs also need support in working with this patient group.

Mainstream health services generally lack an integrated treatment pathway that can support comorbid psychological and physical needs of people who experience MUS (Joint Commissioning Panel for Mental Health, 2016). GPs may be reluctant to communicate to the patient that nothing can be found or that there is no treatment, apart from, if pain is experienced, pain relief and/or pain clinics. After all GPs are trained to diagnose and offer treatment for bodily symptoms, as the first port of call. Possibly due to their training in mainly physical health, the short consultation duration and/or the frequency of appointments, GPs might feel unable to support patients emotionally. Consequently, they may refer the patient to the psychological services (CBT), as the only other option.

Salmon et al. (1999) observe that traditional mental health services have not engaged with people with MUS sufficiently since patients do not see their condition related to anxiety and/or depression, their symptoms being rejected with such a focus. Additionally, a recent practice guideline published by the UK Department of Health (Department of Health, 2014), as a part of the Improving Access to Psychological Therapies initiative, concluded that community mental health teams and primary care mental health services have been unsuccessful in engaging with MUS patients. This they claim is due to patients perceiving their condition as unrelated to mental health problems, so trying to engage them in traditional mental health approaches is usually ineffective.

Despite the mismatch psychological services in the United Kingdom have begun to encompass all patients with MUS although the patient’s priority, the symptom distress, appears to be barely addressed. Instead the focus is on reducing the co-occurring anxiety and depression normally through CBT, the main emphasis of the psychological/mental health service. Despite the service being requested to offer treatment for generic MUS there is only evidence for the effectiveness of graded exercise and CBT for chronic fatigue accompanied with anxiety/depression (Castell et al., 2011).

## Mind-Set of Patients With Mus

In the UK NHS and in western society, the predominant mind-set is that the mind is separate from body (for example, a physical health and mental health service with different structures and budgets). Reflecting this dichotomy patients also see the mind as separate from the body.

Despite the lack of a medical explanation for their symptom (i.e., it does not fit any known pathology) the symptom is nevertheless being physically experienced by the patient. Therefore, logically a physical treatment is expected by the patient which prompts patient and GP to conduct repeated searches for an organic etiology. That having failed the GP has only one choice - to refer to the CBT for any accompanying depression and/or anxiety which does not address the symptom. It is likely that the patient rationalizes their anxiety/depression as being due to the symptom distress rather than the cause of it. Because of the stigma it can feel frightening to the patient to be referred for psychological treatment. It may feel to them as though the medics are saying it is “all in your head” and this is simply not their experience. As we know from research in embodied social cognition (Klin et al., 2003; Gallagher, 2005; Gallese, 2007; Niedenthal, 2007) nothing is solely in the head, the head (brain) is part of the body. In contrast to dualistic thinking the emphasis is the way cognition is shaped by the body and its sensorimotor interaction with the surrounding social and material world. The fields of cognitive psychology (Lakoff and Johnson, 2003; Varela et al., 2017), sociology (Ignatow, 2007), anthropology (Csordas, 1993), and neuroscience (Damasio, 2000; Porges, 2011) recognize embodied experience as a necessity for learning, emotional healing and interpersonal connection. Lakoff and Johnson (2003) work illustrates the mind is inherently embodied, thought is mostly unconscious, abstract concepts are largely metaphorical and cognition is grounded in bodily experience (Lakoff and Núñez, 2000). This new paradigm acknowledges that sensory inputs and motor outputs are integral to cognitive processes.

Therefore, it seems sensible to adopt an embodied approach to learning how to self-manage symptoms for people with MUS, rather than offering any treatment or cure. And starting from where the patient is, i.e., with the experience of the symptom distress in their bodymind and with a patient-acceptable intervention focussing on learning. This is a different approach for engaging people with the mind-set described above as a pre-therapy, which also works for patients who do make connections between body and mind.

## Patient Profile

Steinbrecher et al. (2011) found that MUS made up two-thirds of all reported symptoms with women, younger persons, and non-native speakers, having the highest rates in primary care. Research offers several factors contributing to the development of MUS, and/or associated with MUS, for example:

- Enduring fears and concerns about bodily functions, e.g., hypervigilance toward physical symptoms and perceptions about physical vulnerability;
- Psychosocial factors, e.g., attachment difficulties (Meredith et al., 2008), sexual abuse (Sharpe and Faye, 2006), modeling of functional symptoms (Taylor and Asmundson, 2004), lowered levels of social support (Nakao et al., 2005);
- Psychiatric conditions including depression (Lieb et al., 2007), personality disorders such as borderline and histrionic types (Demopulos et al., 1996). With reference to depression, 70% suffer according to Malhi et al. (2013). This study concludes that collectively somatic symptoms are the most important predictors for determining the severity of depression in primary care and educational initiatives need to focus on depressive subtypes derived from emotional, somatic, and associated symptoms;
- More benefits, hospitalization, GP visits, and unnecessary procedures than people with physical health issues (Fink, 1992; Burton et al., 2012; David and Nicholson, 2012);
- Often have fewer years in formal education (Creed and Barsky, 2004);
- May have had parental neglect or illness in childhood (for women) (Craig et al., 2002);
- Health anxiety/anxiety/panic attacks (Lowe et al., 2008);
- Generally, have more sick leave (Kisely et al., 1997; Aamland et al., 2012);
- Are more likely to be unemployed (Hiller et al., 2003);
- Comparable to medically explained conditions in their impairment of physical function but have a considerably poorer quality of life than medically explained conditions (Smith et al., 1986);
- An association has been identified between somatization and alexithymia in a large, national representative sample in Holland (Mattila et al., 2008);
- Sometimes there is past or current family dysfunction and/or a history of trauma or abuse, (Sharp and Harvey, 2001; Fiddler et al., 2004), particularly for women for some symptoms, for example gastrointestinal (Van Tilburg et al., 2010);
- Some insecure attachment styles have been correlated with MUS, for example avoidant/dismissive (Adshead and Guthrie, 2015).

Patients with MUS are distressed because they have long term and over-whelming bodily symptoms (frequently more than one) without a medical explanation. Additionally, they may feel desperate because no one appears to be able to support them to manage their experience. Not surprisingly they are both anxious and depressed and these feelings exacerbate the experience of the symptoms, thus they can go into a downward spiral and feel out of control – an unhelpful feedback iteration. Patients may feel isolated often because they believe they are the only one for whom their GP cannot find an explanation and abandoned because family and friends cannot bear to listen anymore. Consequently, they may feel out of control and that no one can help them.

Approaches which target the internal world of feelings, perceptions and link these to behavior may be beneficial to change processes in MUS. One study concluded that directly observing the physical effects of emotional experiencing in MUS provides sensory evidence which enables patients to make mind–body connections (Town et al., 2017).

There can be a lack of support for patients to self-manage due to, for example GPs being unable to support a patient where the cause of their symptoms is unknown and/or GP’s lack the time to devote to such issues.

The novel intervention described below, The BodyMind Approach (TBMA), is not a panacea and it does not replace GP’s judgment about the necessity for further investigations hence it is not an “either or” but an “and/both” intervention if required in the meantime. As one GP commented “it can do no harm” and when successful reduces costs for the UK NHS Clinical Commissioning Group.

## The Bodymind Approach

### Theoretical Underpinnings

The BodyMind Approach is a newly developed intervention to overcome the obstacles of the patient mind-set and lack of treatment option. It is a specialist, community-based program for primary care patients with MUS based on research (Payne and Stott, 2010) and practice-based evidence (Payne, 2015, 2017a; Payne and Brooks, 2016, 2017) conducted at the University of Hertfordshire, United Kingdom. During the research a cost effectiveness study was conducted by a health economist to demonstrate the expected savings to primary care of implementing TBMA (Payne and Fordham, 2008). The subsequent delivery in the NHS was through a spin-out body from the University transferring knowledge for impact (Payne, 2017c).

Many MUS patients who have different mind-sets require different interventions to those currently on offer. TBMA, as a different intervention, engages patients by working directly with their symptoms rather than from the mental faculty. TBMA addresses this resistance to engagement because it is framed as a learning approach not a treatment or psychological therapy. However, once engaged in a process this can promote feelings of control and self-management.

The BodyMind Approach is a research-informed, practice-based evidenced model designed specifically for assisting people with MUS to gain the learning required to monitor and effect their perceptions, emotions, thinking, and behavior to self-manage their symptom/s to maintain a satisfactory quality of life despite the symptoms. Consequently, it differs from other approaches which are standardized for mental health treatment and healing in general, such as CBT.

The BodyMind Approach has been developed from enactive, embodied psychotherapy (dance movement psychotherapy) adapted specifically as a learning tool for the MUS population. TBMA engages educationally with participants. Experiential learning is key to the process of feeling in control which can be empowering and encourages resilience to sustain self-management. This process then becomes a virtuous circle and changes habits building new habits. Significant features of the symptom, the effect on feelings and functioning and the relationship of related behavior and thinking are explored through creative arts expression leading to learning and then realistic goal setting by the participant. The learning experience begins by engendering an attitude that change is needed, raising this awareness is central to the process whereby participants also need to be helped to engage as an equal in the desire for change. They learn to take responsibility for the management of their symptom/s. Participants acquire new skills, understanding, and knowledge on how to change the way they behave toward, feel, perceive, and experience their symptoms. This learning is consolidated to reinforce the change through an individualized, participant-centered, tailor-made action plan followed by the participant for 6 months post-group.

Underpinned by phenomenology and recent neuroscientific research, the embodiment paradigm focuses on the implicit functioning of the body in perception and performance (Merleau-Ponty, 1962; Salmon et al., 1999; Fuchs and Schlimme, 2009; Koch and Fuchs, 2011; Fuchs, 2012; Fuchs and Koch, 2014). The “lived body” is understood as a background to our experience of the world. Organizing our pre-reflective sense of self and agency allows us to attune to the environment and to others through a shared intercorporeality (Fuchs and Schlimme, 2009; Payne, 2017b), an aspect of intersubjectivity. In TBMA body, mind, action, and perception is understood as a unity, and acknowledges the need to target body experiences to change emotions and behavior. It does not require the use of language but may stimulate participants to explore their symptom/s and feelings in a variety of ways which may or may not include language. Following Bateson’s concept of the “embodied mind” (Bateson, 1972, p. 317) and Varela et al. (2017) proposed an alternative to the dominant cognitivist tradition – an embodied and enactive approach. This suggests that cognitive processes cannot be confined in the brain but are formed and influenced by the whole-body system interacting with the environment. Brains and minds are embodied, and our bodies are embedded in the world. This new conceptualisation has spread into psychology and cognitive science which has implications for education, learning, social cognition, approaches to clinical practice, therapy, and change processes. According to the enactive stance (the mind cannot be understood a separated from the body) actions and movements perform an essential part in meaning-making. Through our movement we enact a world of meaning and self-generate our identity in the process (Galbusera and Fuchs, 2013).

The BodyMind Approach explores the experience of the symptom by working from the body to the mind (Lakoff and Johnson, 2003; Varela et al., 2017), in this way it honors both conscious and unconscious processes. Sensation, perception, emotion, and cognition are integrated. This is achieved in a facilitated group by using creative, embodied practices. Relationships within the group are emphasized learning with, and from, each other. Practices which “bear the symptom in mind” such as body awareness through mindful expressive movement, dialoguing with the symptom through drawing and speaking, mindfulness, progressive relaxation, and breathing are suggested. Through such practices people gain improved emotional self-regulation via an understanding of their symptom and making meaning of its nature, characteristics, purpose and the role it plays in their lives. Consequently, they may be more able to make conscious decisions about how they change their lives to manage their symptoms.

Cognition is a dynamic sensorimotor interaction expressed through bodily activities. Thelen and Smith (1994) apply dynamic systems theory to developmental psychology. They suggest behavior results from the interaction between the body action and changing environmental contexts. They see development as an emergent and self-organizing product of many decentralized and local interactions taking place in real time. From this view the sensory experience of the symptom is a product of the interaction between the internal and external environment and is thus self-organizing and emergent (Siegal, 2017). If this is so, then changing the internal and/or the external environment can lead to change in the experience of the bodily symptom and vice versa. There is some evidence showing TBMA is helpful because it creates a new environment from which participants can change their perceptions and then choose to change their behavior. TBMA stresses change in both the internal and external environment. The internal is addressed through creative body-based practices whereby the symptom could be the metaphor for the self-narrative (Gallagher and Hutto, forthcoming). The external setting is engaged with by the participant through others acting as witnesses and from the facilitator’s holding presence. This approach is based on the integration of dance movement psychotherapy (Chaiklin, 1975; Siegel, 1984; Stanton-Jones, 1992; Meekums, 2002; Payne, 2003, 2006a, 2017b), authentic movement (Chodorow, 1991; Whitehouse, 1999; Adler, 2002; Payne, 2006b), bodymindfulness, and the arts. The theory is that the body pre-occupation is the foreground and the mind the background, which enables the symptom to act as a gateway to the mind, i.e., “playing with the symptom so it does not play on you” as one participant said.

The BodyMind Approach is based on the functional unity of mind and body and recognition that our psychological experiences are formed, experienced, expressed, and reshaped through the body. It is a bio-psychosocial approach because it is holistic and integrates body (bio) and mind (psyche) in a group (social) setting. Furthermore, it encompasses the participant’s personal social situation when they are exploring their symptoms in TBMA.

It employs a behavior change model whereby a different perception of the body (and the symptoms) is gained. It becomes possible for the participant to reframe symptoms as an ally (a protective factor to buffer any effects of stress) rather than the enemy. Hence, a conscious understanding of the symptom can inform the person when they are out of balance, so helping them to take steps to re-balance (mindfully listening to and valuing the body and its signals). Thus, there is no need to attempt to banish the symptom but to welcome it as an early warning system for self-care action, i.e., to be able to cope. This is an empowering experience enabling the participant to take back control. The focus is on living well with symptoms rather than a cure.

As TBMA includes the body it is termed a “bottom up” approach rather than the conventional top-down cognition orientation which privileges language for a set of beliefs and emotions. Neuroscience shows us that cognitions and emotions are embodied (Shapiro, 2011). According to Shapiro (2011) concepts partially originate in a subjective emotional experience anchored in the body. They are then simulated by the activation of related aspects of those experiences, for example, current trauma resonating with a previous trauma.

The unconscious and/or conscious body pre-occupation found in people with MUS, is used to prompt curiosity about symptoms. The sensory perception and physical impulses expressed as movement, in whole or parts of the body, helps gain access to the roots of the previous experience in the lived body. That is, to the automatic impulses and the pre-lingual processes.

### Philosophy

The BodyMind Approach promotes wellbeing and facilitates the recovery model in mental health (Ramon et al., 2007). This involves developing hope, a secure base and sense of self, supportive relationships, empowerment, social inclusion, coping skills, and meaning-making. It espouses the idea that positive change is possible. This promotes hope which is a powerful message for people feeling so dejected from their experience. Integral to the program is the acceptance of the participant’s symptoms and the belief that they are real, not all in the head, honoring the participant’s lived body experience. Symptoms can be understood and worked with to learn to live well with them.

The program emphasis the non-medical aspect and normalizes rather than pathologises MUS which can help to alleviate anxiety. CBT and psychotherapy fail to normalize. For example, in TBMA sessions are termed “workshops” (not treatment or mental health) are full of other “participants,” (not patients) experiencing MUS and held in a “community venue” (rather than GP practice or mental health/wellbeing center) leading to a de-medicalization of the body.

The focus is on the explanatory model emphasizing the inter-relationship between body and mind as a part of normal human experience. It is the lived body which is the focus. The working model employs acceptance of the symptom by the participant which may help control symptom distress. Many participants report that this results in reduced symptom distress. The aim of TBMA is to promote self-management so participants can live well with their symptoms day-to-day so, as one participant explained, “the bad days are not so bad any more.” MUS participants come with a lack of confidence, downtrodden, and feeling inept. They feel disempowered as a result of the time spent in the health system searching for an explanation which cannot be found.

Additionally, belonging to a group with other people with MUS also helps to normalize their experience and reduces the tendency to catastrophize. It is important for participants to be nurtured toward taking responsibility for their body and it’s functioning rather than expecting others to provide a fix. Up to this point the participant’s experience of their body has often been that it has been treated as an object to be fixed by the medics. The participant has internalized this message and expects the medics to solve their symptom distress. TBMA encourages an internal locus of control, whereby participants begin to feel empowered to manage their symptoms without medical intervention. TBMA helps participants to value their internal subjective bodily experience and to use this to promote emotional self-regulation, self-reliance, and resilience, rather than seeing their body as an object. They are a “bodymind,” participants say they experience improved connections between their body and mind, rather than “having a body.” This is a change in both perception and action. Bodily symptoms such as those in MUS and the co-occurring depression and/or anxiety can be understood as resulting from an inability to emotionally self-regulate. The default position when stress occurs becomes located in the dysfunctional bodily symptom. Therefore, this becomes an iterative aggravation of the symptom itself leading to a downward spiral. Hence, the measurement tools employed in TBMA need to assess levels of symptom distress, wellbeing (MYMOP2); functioning (GAF); depression (Phq9) and anxiety (GAD7). They either are, or act as, proxy measures of emotional self-regulation.

The BodyMind Approach employs a variety of ways of knowing. One way of knowing is through cognition, thinking about things as in CBT. Another way is hearing myself speak about things. Yet another way of knowing is physically feeling myself act/move in response to conscious or unconscious thoughts, feelings or sensation. A different way of knowing is seeing another person move/act and noticing any sensations, images, stories/thoughts/interpretation, feelings which are elicited in me by witnessing them move with their symptom in mind. TBMA therefore becomes a tool for exploring mind and body identity in a relational and an integrated way. These practices help people to listen to the meaning they gives to their bodymind experiences, connecting to their personhood in a less stigmatizing way driven by dualistic assumptions.

A recent personal construct psychology study with MUS participants (*N* = 20) found symptom constructs were well integrated within mind-body construct systems of participants, possibly supporting the notion of “enmeshment” of self with symptoms (Pincus and Morley, 2001). The way in which the self in general is perceived relative to times when symptoms are worst appeared to be particularly important for participants. Perceiving a dissimilarity between self in general and self when symptoms are at their worst reduced anxiety. This discrepancy did not correlate with symptom-severity scores suggesting it is the perceived difference, rather than the absolute difference made by symptoms, which effects anxiety again supporting an enmeshment-based formulation of MUS. If a number of undesirable characteristics are enmeshed with symptoms, construing these negative differences as residing more in the self when symptoms are worst, then the self in general may serve to protect from construing the self as having globally changed in undesirable ways (Hellstroem, 2001).

### Terminology

Terminology is extremely important and needs to be informed by the audience, for example, participant and GP perspectives, mind-sets, and language. Originally, in the pilot study, participants were invited to comment on the group’s title, i.e., initially termed dance movement psychotherapy, it morphed into “learning group” then a “symptoms group,” and now the name “living well” group is under consideration to ensure acceptability of the title for participants. With participants the intervention is referred to as a “course.” The terms have changed as the learning developed.

In the original study GPs were invited to focus groups to elicit their views on terminology and the content (Payne et al., 2009, Unpublished). Terms found to be acceptable to GPs were “The Symptoms Clinic” or “The MUS Clinic.”

### Structure of the Program

Medically unexplained symptoms may develop in childhood as a strategic response to adversity and attachment difficulties with caregivers (Crittenden, 2006; Waldinger et al., 2006; Kozlowska, 2007; Roelofs and Spinhoven, 2007; Anderson et al., 2013). Aspects of self and identity which could be threatened in MUS may be those which are embodied and not easily verbalized. Insecure attachment style is often displayed by such a patient population (Taylor et al., 2012; Adshead and Guthrie, 2015).

A significant association was found between insecure attachment style and frequent attendance, even after adjustment for sociodemographic characteristics, presence of chronic physical illness and baseline physical function. The association was particularly strong in those patients who believed that there was a physical cause for their initial MUS Taylor et al. (2012, p. 855).

The structure of the program is designed to take account of this insecure attachment style in the following ways.

- There are 12 × 2-h group workshops emphasizing the group process and giving time for change.
- Front loaded for the first four sessions which are two per week to accelerate the feeling of safety with the facilitator and other group members. This intensive phase reduces drop-out rates and ensures group cohesion occurs.
- Individual meetings with a clinical psychologist and group facilitator before the group to promote safety and give familiarity with the assessment process and the group facilitator.
- Thereafter there are eight further group sessions to develop and extend the group experience and promote further change this is the equivalent in the performing phase as in forming, storming, norming, and performing (Tuckman, 1965). After the 12 group workshops there is another individual meeting with the group facilitator and the clinical psychologist for saying goodbye and developing the action plan and to undertake a further assessment.
- The second phase is 6 months of non-face to face contact during which time participants enact their individual action plan and are contacted to let them know they are not forgotten and keep them on track with their action plan through text, email, and letters.
- The total program duration lasts for 9 months from referral, due to the time required for participants with such chronic symptoms to learn to self-manage. The constant connectivity with the participant helps relieve anxiety commonly found in insecure attachment style.

The complexity and long-standing nature of the symptoms means that it is very difficult to address symptoms without sufficient time. Total face to face contact time in TBMA is 27 h with the group facilitator, 1.5 h with the clinical psychologist and with non-face to face facilitator contact every 6 weeks. This far outweighs the time allowed for currently available CBT which is an average of only six sessions.

The structure of working in a group also helps to address the feelings of isolation and abandonment that many MUS patients have experienced, and the facilitator may perform the role of an attachment figure during the early stages until the participants self-confidence has increased.

### Learning to Self-Manage

Self-management involves the principles of adult learning, whether combined or not with biological, psychological, and social interventions, treatments or techniques. The overall aim is to maximize the emotional self-regulatory function of the individual patient. Empowering people to be confident in their ability and capacity to care for themselves reduces the impact of the condition on day-to-day functioning and prevents the impact increasing.

A Cochrane Collaboration Review examined the more rigorously tested interventions to improve primary care for diabetes, another long term chronic condition, and included the conclusion that patient-oriented interventions of an educational or supportive nature were amongst successful approaches (Renders et al., 2001). This confirms earlier literature that chronic disease interventions positively affecting patient well-being necessarily include systematic efforts to increase patients’ knowledge, skills, and confidence to manage their condition (Von Korff et al., 1997). Traditional patient education emphasized knowledge acquisition and didactic classroom teaching. While such interventions increased knowledge, they were unsuccessful in changing behavior or improving disease control and other outcomes (Clement et al., 2000). Research has shifted the focus toward not only improving patient’s knowledge of their condition, but also confidence and skills in managing it (Norris et al., 2001). This research reinforces the patient’s crucial role in managing the condition, helping them to develop reasonable goals for improving their self-management, to identify any barriers to this achievement and designing a plan to carry out actions to reach those goals. Supportive reminder systems to reinforce the plan are also recommended (Woolf et al., 1999). There is evidence that individual and group interventions emphasizing peer contribution, patient empowerment and the acquisition of self-management skills are effective in diabetes, asthma, and other chronic conditions (Gibson et al., 2001). Furthermore, patients need to learn to manage the complex psychosocial issues arising from their condition. Thus, self-management may be one of the main ways of closing the gap between patient needs and health service capacity (Barlow et al., 2002). Emotional self-regulation (Barlow, 2001) is crucial to resilience, life will continue to generate stresses for patients who may experience their symptoms even more as a result if they cannot manage stress effectively. Learning about the possible stress responses as they occur in the bodymind can be helpful to understand their bodily reactions.

Educational interventions have been commonly used as strategies to improve health outcomes of patients with low health literacy (Schaefer, 2008). Studies have found health education may improve patients’ knowledge and treatment of a disease leading to better treatment adherence and patients taking a more positive role in the management of their health (Shaw and Bosworth, 2012). Additionally, changes to lifestyle and increased adherence to antihypertensive medications to improve effective blood pressure control in hypertensive patients have been found (Meyer et al., 1985). Recent research concluded interactive education workshops may be the most effective strategy in community-based health promotion education programs for hypertensive patients in improving patients’ knowledge on hypertension and alleviating clinical risk factors for preventing hypertension-related complications (Lu et al., 2015). Consequently, it can be argued that an interactive, workshop learning model may be helpful in supporting patients with MUS to self-manage their condition.

The BodyMind Approach offers just such a model, informed by pedagogical roots in adult learning and teaching, transformational and life-long learning, self-directed learning and knowledge sharing at its heart. In TBMA the learner is actively involved in identifying their goals and problem-solving to reach them via an individualized action plan for self-management. Self-responsibility is encouraged, and self-directedness is inherent in the patient setting relevant goals and learning how to manage symptoms as a result of their learning from the various practices offered during the group workshops. Learners are actively facilitated to learn to manage/control their symptoms. TBMA includes the learner’s lived experience of their bodily symptoms, from which needs arise leading to goals being identified. The facilitated group environment provides a safe place for two vital elements for learning and new skill acquisition to take place. Firstly, there is ability to experiment with new ways of being in the body. Secondly, there is the important element of practicing and gaining confidence to employ the bodily changes. Together these may lead to the dynamic of thinking differently about the body and the drive to practice even more, ultimately leading to a virtuous cycle of improvement.

Evaluation takes place at the end of the group workshops and as the learner reflects in the group, with the facilitator and in her reflective learning journal the capacity for self-direction is stimulated supporting transformational learning (Mezirow, 1997). The pathway for each learner is individual. Life experiences, beliefs and lifestyle in relation to perceptions of symptoms are evaluated together from which transformational learning can occur. There is a focus on problem-solving in the context of the real, body-felt world of the patient. The objectives of the group depend on the themes and issues arising in the group at any one time as perceived by the facilitator, although a manual has been designed to support the facilitator in activities.

Participants learn to take responsibility and develop confidence in their capacity to take appropriate action to resolve stressful situations in a changing environment. They learn to be openly communicative, creative, and flexible as well as to incorporate more positive values. Participants are given home practice, so they learn the strategies can work for them, and are reproducible in different situations. This supports them once the group has ended and the 6 months phase two helps them to stay on track with their action plan.

### Content of Program

The BodyMind Approach uses the creative arts, for example, expressive movement, drawing, clay-making, expressive writing, are used to explore the symptom, to make meaning from it which in turn helps with the sense of control. This facilitates emotional regulation, bringing the bodymind back into balance and wellbeing leading to self-management. The creative arts may be used as the catalyst for developing relationships in the group and as symbolic and metaphoric representations of their inner worlds. Risk-taking and experimentation affordable through the arts in a facilitated, supportive group can enhance emotional self-regulation. Attunement with another develops presence (being in the here-and- now) as in authentic movement dyads whereby initially words are secondary to the non-verbal communication. Feelings, images, sensations, and thoughts communicated through movement are then processed verbally together with a witness can further support emotional self-regulation. Self-attunement as in bodymindfulness practices, such as walking mindfully can also support emotional self-regulation. Furthermore, the kinesthetic-sensory qualities of art, clay, and expressive movement/dance, including tactile, synchrony, entrainment, and rhythm, can mediate lower brain functions such as heart rate and respiration. The arts have unique sensory qualities which can meet with the sensory experiences in the body (e.g., symptoms). It is not solely the use of creative arts that is important but the fact they are employed in a group setting whereby participants make sense of symptoms with each other.

A participant saw an image of a lion emerging from sensing her symptom which she interpreted as anger. This helped her to consider how to moderate her anger which tended to trigger her symptom. The participant dialogs with their symptoms to explore and better understand, re-frame or gain an explanation of meaning, origins, triggers, and maintenance of them day-to-day. Progressive relaxation, raising awareness of body signals linked to the symptom, self-care, breathing, body awareness practices, and inter-relational exercises are delivered by working in twos, threes and as a group. Body awareness is increased by movement and other sensory practices to detect signals coming from the body to alert the individual when they are feeling a stress-response. On becoming aware of these responses, the individual can take appropriate action (for example, slowing down breathing) to mediate them. One participant learned how to breathe correctly, i.e., from and into the abdomen, and discovered this method when practiced regularly relieved her symptoms almost entirely, releasing energy so she could “dance around the kitchen.”

The BodyMind Approach is a profoundly innovative approach aiming to connect bodily states with emotional and cognitive elements through enactment and expressive movement. TBMA does not explicitly refer to any underlying psychological conflict or to identify and attempt to modify dysfunctional thinking patterns.

### Referral Criteria

Referrals are from GP and self-referral and criteria are based on those in the previous research study (Payne and Stott, 2010). Inclusion and exclusion criteria are vital to the referral system which is via GPs and self-referral. Inclusion criteria:

- 18+ years;
- MUS diagnosis for at least 6 months;
- Frequent attender (more than four visits per annum);
- Presentation for more than 6 months;
- Co-morbid depression/anxiety;
- Fluent English speaker.

Exclusion criteria:

- Current relevant physical health problems;
- Fewer than 4 GP consultations in previous year;
- Trauma in previous 6 months;
- Current relevant physical disability;
- Complex bereavement previous 6 months;
- Learning disability;
- Primary diagnosis of psychiatric condition in previous 6 months;
- Current substance misuse;
- A diagnosed eating disorder.

### Assessment Tools and Procedure

We define self-management as the ability to live well with symptoms, especially at times of stress, achieved through emotional self-regulation. Tools for measuring emotional self-regulation do not appear to focus on the link between the brain-body and emotion. Emotions are based in, and expressed through, the body and movement. Accessing the body is a way in to accessing emotions (Michalak et al., 2009). Emotional regulation is fundamental to physical and psychological health, that is wellbeing (Aldao and Nolen-Hoeksema, 2012). In TBMA, practices which support emotional regulation include controlled breathing (Philippot et al., 2002); bodymindfulness and progressive relaxation (reduces anxiety) (Manzoni et al., 2008) together with movement. Consequently, emotional regulation is not merely a mental process but the result of an interplay between the body and mind. “Sensory-motor processes are not just side effects, but rather are vital in instantiating and regulating a desired emotional state, and thus to the effectiveness and efficiency of emotion regulation” (Veenstra et al., 2017, p. 1374).

To evaluate the outcomes of the intervention with reference to self-management, we selected proxy measures of emotional self-regulation using standardized tools. The measurement tools employed to assess changes in emotional self-regulation were PhQ9 for depression; GAD-7 for generalized anxiety; GAF for general functioning; MYMOP2 for symptom distress and wellbeing; and data from these are collected and analyzed at three time points, pre-course, post-course and at 6 months follow up, according to reliable change criteria. Furthermore, a questionnaire collects data on demographics belief systems, employment status, social and leisure pursuits, and GP/hospital visits. The combination of the above measurement tools is unique. MYMOP focusses on wellbeing, the symptom distress and activity. Participants select an activity which their symptom prevents them from performing. The level to which they are able to perform this activity acts as a baseline measure for future data comparison. MYMOP, however, is a measure on which it is relatively easy to show improvement. Nonetheless since participants are preoccupied with their symptom distress it is the most relevant aspect to measure. The co-occurring depression and anxiety are measured as these are features in MUS but not necessarily the over-riding concern of the participant, and often result from the symptom distress. Assessment is conducted in the week prior to the course commencing via a telephone interview with a clinical psychologist. The post-course assessment is conducted in the week the course ends and the follow up is 6 months later.

### Facilitation

All facilitators have a Masters in an embodied psychotherapy, e.g., dance movement psychotherapy, creative arts therapies, body psychotherapy and with at least 5 years of group work with adults. They undergo a training over 4 days in TBMA in group work for participants with MUS and are assessed and certified as facilitators thereafter. The facilitator is the catalyst for change constantly responding to the needs of the group in the moment using reflection-in-action as a result of their professional judgment, experience, and training. Participants feel seen and understood and supported in a kind and caring manner.

According to participant feedback the facilitator is a crucial part of the treatment process. Many facilitators also have (or have had) an MUS which give them greater understanding and empathy of the participant experience. A manual has been designed as a tool to support the delivery of TBMA with examples of practices, sessions, and theoretical background. It is not a recipe book, nor a minute by minute prescription for sessions, rather it allows for professional judgment and the needs of each group to be taken into account when delivering sessions. Thus, the group sessions are not standardized and could be criticized to some extent regarding program integrity yet would score highly on responsiveness. Nevertheless, there is a range of themes which are expected to be covered during the course of treatment which have been previously identified from facilitator and participant feedback. Groups have been delivered by several different trained facilitators all of which have shown similar positive outcomes. This indicates that it is the intervention rather than the facilitator which is having the effect. This does not mean the facilitator is unimportant, indeed participants report that the facilitator is crucial. However, the results do not rely on a single person or a rigid formula.

### The Power of the Group

The program follows a group work model to support a sense of belongingness to reduce isolation often felt by this population. It enables people to meet others with MUS, often for the first time. They share similar experiences of the NHS and family and friends. They frequently have similar thoughts and feelings in relation to the unknown threat, such as wondering if they have the big “C” or some incurable disease. The facilitated group setting provides for motivation for change, peer support, reduction in isolation and making new and long-lasting friends.

The group support is usually noted in participant feedback as highly valued. The richness offered by a facilitated group brings in new and interesting ideas, solutions, and coping strategies.

The group is heterogeneous, comprised of people with a variety of symptoms. TBMA assumes that there is a single generic underlying reason for this range of symptoms, such as fibromyalgia, irritable bowel syndrome, headache, chronic fatigue, chronic pain, etc., whatever that reason may be, and this leads to the inclusion of all symptoms in one treatment group. We do not know the cause of the symptoms, and neither do most participants, but the approach appears to work with a variety of origins, for example, trauma, family ill health, insecure attachment styles, complex bereavement, sexual abuse, neglect, etc. The cause being unknown does not appear to matter because TBMA is not proposed as a psychotherapy *per se* to uncover causes, rather working with the presentation/effects in the lived body.

### Action Planning

During the groups participants are provided with a personal journal in which they are encouraged to write/draw each session. Based on their new learning from the experience of the group sessions in the final session they are invited to design an action plan for change. This becomes the template for action in the subsequent 6 months after the sessions end. The plan needs to be realistic and to support small changes in the way they manage their life/symptoms. They meet with the facilitator individually to review the plan. Six weeks later they receive a self-written letter in the post-delineating their action plan and how they will adhere to it. Twelve weeks later the facilitator writes to them to remind them of the steps which they were expected they would make to enact the plan. This system encourages the new habit to become embedded and embodied for sustainable change to take place. At 18 weeks post-course a text and email are sent asking how they are doing. At 6 months another assessment (follow up) is conducted.

## Conclusion

We would agree with Henningsen et al. (2007) active participation of patients in treatment approaches involving for example, exercise and psychotherapy, seem to be more effective than those that involve passive physical measures. TBMA reflects the finding from that review. By integrating health psychology with health education and training through TBMA psychologically aware and psychologically resistant people with MUS in primary care can be engaged over approximately 9 months to self-manage. Over and above this engagement participants have reported they enjoy the groups and have benefited from them. There is a need to offer a range of interventions for this complex population in primary care of which TBMA is one.

## Author Contributions

HP conceived the idea and developed the model from dance movement psychotherapy. SB supported the development of this article with input from adult learning theory and personal experience with medically unexplained symptoms.

## Conflict of Interest Statement

The authors declare that the research was conducted in the absence of any commercial or financial relationships that could be construed as a potential conflict of interest.
