# Corrigendum: Different Strokes for Different Folks: The BodyMind Approach as a Learning Tool for Patients With Medically Unexplained Symptoms to Self-Manage

**DOI:** 10.3389/fpsyg.2019.01837

**Published:** 2019-08-07

**Authors:** Helen Payne, Susan Brooks

**Affiliations:** School of Education, University of Hertfordshire, Hatfield, United Kingdom

**Keywords:** medically unexplained symptoms, primary care, embodied approaches, adult learning, self-management, metaphor, symbol, group

In the original article, there was an error regarding the cost of medically unexplained symptoms (MUS).

A correction has been made to the Abstract:

“Medically unexplained symptoms (MUS) are common in both primary and secondary health care. It is gradually being acknowledged that there needs to be a variety of interventions for patients with MUS to meet the needs of different groups of patients with such chronic long-term symptoms. The proposed intervention described herewith is called The BodyMind Approach (TBMA) and promotes learning for self-management through establishing a dynamic and continuous process of emotional self-regulation. The problem is the mismatch between the patient's mind-set and profile and current interventions. This theoretical article, based on practice-based evidence, takes forward the idea that different approaches (other than cognitive behavioral therapy) are required for people with MUS. The mind-set and characteristics of patients with MUS are reflected upon to shape the rationale and design of this novel approach. Improving services for this population in primary care is crucial to prevent the iterative spiraling downward of frequent general practitioner (GP) visits, hospital appointments, and accident and emergency attendance (A&E), all of which are common for these patients. The approach derives from embodied psychotherapy (authentic movement in dance movement psychotherapy) and adult models of learning for self-management. It has been developed from research and practice-based evidence. In this article the problem of MUS in primary care is introduced and the importance of the reluctance of patients to accept a psychological/mental health referral in the first instance is drawn out. A description of the theoretical underpinnings and philosophy of the proposed alternative to current interventions is then presented related to the design, delivery, facilitation, and educational content of the program. The unique intervention is also described to give the reader a flavor.”

Additionally, a correction has been made to the Introduction, paragraph one:

“Medically unexplained symptoms (MUS) are a thorny issue in primary care. Despite the differing nomenclature, the recent DSM-5 terms it as somatic symptom disorder (SSD) but is yet to achieve general usage. Many general practitioners (GPs) appear to reliably recognize MUS without the need for standardized assessments (Rasmussen et al., 2008). This population present with many, various and nebulous physical and psychological ailments (Rosendal et al., 2005) and constitute more than 25% of all new hospital and GP appointments (Fink et al., 1999; Reid et al., 2001). In England MUS has been estimated to cost £3 billion in 2008–2009 rising to £18 billion if loss of productivity, benefits and quality of life are accounted for Bermingham et al. (2010).”

The authors apologize for this error and state that this does not change the scientific conclusions of the article in any way. The original article has been updated.

## References

[B1] Bermingham S. Cohen A. Hague J. Parsonage M. (2010). The cost of somatisation among the working-age population in England for the year 2008-09. Mental Health Fam. Med. 7, 71–84. PMC2939455 22477925

[B2] Fink P. Sorensen L. Engberg M. Holm M. Munk-Jorgensen P. (1999). Somatization in primary care: prevalence, healthcare utilization & GP recognition. Psychosomatics 40, 330–338. 10.1016/S0033-3182(99)71228-4 10402880

[B3] Rasmussen N. H. Agerter D. C. Colligan R. C. Baird M. A. Yunghans C. E. Cha S. S. (2008). Somatization and alexithymia in patients with high use of medical care and medically unexplained symptoms. Mental Health Fam. Med. 5, 139–148. PMC2777575 22477862

[B4] Reid S. Wessely S. Crayford T. Hotopf M. (2001). Medically unexplained symptoms in frequent attenders of secondary health care: retrospective cohort study. BMJ 322:767. 10.1136/bmj.322.7289.767 11282861 PMC30552

[B5] Rosendal M. Oleson F. Fink P. (2005). Management of medically unexplained symptoms. Br. Med. J. 330, 4–5. 10.1136/bmj.330.7481.4 15626783 PMC539826

